# Implementation of a quality improvement strategy to increase outpatient kidney transplant referrals

**DOI:** 10.1186/s12882-020-01855-0

**Published:** 2020-05-20

**Authors:** Samira S. Farouk, Sara Atallah, Kirk N. Campbell, Joseph A. Vassalotti, Jaime Uribarri

**Affiliations:** Division of Nephrology, Department of Medicine, Icahn School of Medicine at Mount Sinai, Box 1243, One Gustave L. Levy Place, New York, NY 10029 USA

## Abstract

**Background:**

Kidney transplantation remains the optimal therapy for patients with end stage kidney disease (ESKD), though a small fraction of patients on dialysis are on organ waitlists. An important barrier to both preemptive kidney transplantation and successful waitlisting is timely referral to a kidney transplant center. We implemented a quality improvement strategy to improve outpatient kidney transplant referrals in a single center academic outpatient nephrology clinic.

**Methods:**

Over a 3 month period (July 1–September 30, 2016), we assessed the baseline kidney transplantation referral rate at our outpatient nephrology clinic for patients 18–75 years old with an estimated glomerular filtration rate (eGFR) of less than 20 mL/min/1.73m^2^ (2 values over 90 days apart). Charts were manually reviewed by two reviewers to look for kidney transplant referrals and documentation of discussions about kidney transplantation. We then performed a root cause analysis to explore potential barriers to kidney transplantation. Our intervention began on July 1, 2017 and included the implementation of a column in the electronic medical record (EMR) which displayed the patient’s last eGFR as part of the clinic schedule. In addition, physicians were given a document listing their patients to be seen that day with an eGFR of < 20 mL/min/1.73m^2^. Annual education sessions were also held to discuss the importance of timely kidney transplant referral.

**Results:**

At baseline, 54 unique patients with eGFR ≤20 ml/min/1.73 m^2^ were identified who were seen in the Clinic between July 1, 2016 and September 30, 2016. 29.6% (16) eligible patients were referred for kidney transplantation evaluation. 69.5% (37) of these patients were not referred for kidney transplant evaluation. 46.3% (25) did not have documentation regarding kidney transplant in the EMR. nephrologist’s most recent note. Following the intervention, 66 unique patients met criteria for eligibility for kidney transplant evaluation. Kidney transplant referrals increased to 60.6% (*p* <  0.001).

**Conclusions:**

Our pilot implementation study of a strategy to improve outpatient kidney transplant referrals showed that a free, simple, scalable intervention can significantly improve kidney transplant referrals in the outpatient setting. This intervention targeted the nephrologist’s role in the transplant referral, and facilitated the process of patient recognition and performing the referral itself without significantly interrupting the workflow. Next steps include further investigation to study the impact of early referral to kidney transplant centers on preemptive and living donor kidney transplantation as well as successful waitlisting.

## Background

Chronic kidney disease (CKD) is a common and complex disease affecting approximately 37 million American adults [1]. CKD is rising in prevalence with high public health costs and is associated with a high degree of morbidity and mortality [2, 3]. Kidney transplantation remains the optimal treatment for patients who progress to end stage kidney disease (ESKD). Unfortunately, only approximately 14% of patients on dialysis are waitlisted for kidney transplantation [4] – several barriers to timely kidney transplantation have been described, including medical and financial barriers [5, 6], racial and ethnic disparities [7], and no identified living donor or donor evaluation delays [5]. Not surprisingly, both earlier timing of and presence of referral to kidney transplantation have been identified as predictors of preemptive kidney transplantation [5].

The National Kidney Foundation’s Kidney Disease Outcomes Quality Initiative guidelines emphasize patients who reach CKD stage G4 should receive timely education about kidney failure and options for its treatment, including kidney transplantation as part of nephrology consultative services [8]***.*** Referral from an outpatient nephrology clinic or dialysis unit to the transplant center is a critical initial step toward successful kidney transplantation, and transplant education in early stages of CKD may increase rates of pre-emptive kidney transplant [5]. Observational studies demonstrate factors associated with low referral and assessment rates for kidney transplantation include racial and socioeconomic disparities, geographic factors, local transplant center density, and size as well as ownership of the referring dialysis facility [9–12]. Most of the literature regarding advanced CKD referral for transplant has focused on variations in the duration of nephrology care before ESKD [1, 13]. Thus, the process of kidney transplantation referrals from CKD nephrology clinics is understudied. Timely referral to the transplant center presents an opportunity to not only educate patients about the kidney transplant evaluation, surgical procedure and care, but also to potentially avoid dialysis by increasing access to pre-emptive transplant. Observational data demonstrate that the pre-emptive kidney transplant population has improved outcomes relative to patients that are transplanted after initiation dialysis even after accounting for lead-time bias [8, 14, 15]. Further, early adult nephrology referral has been associated with improved dialysis preparation and mortality as well as decreased hospitalization rates [16]. In children with CKD, earlier nephrology referrals have been shown to lead to a later initiation of dialysis than those who were referred later [17] .

The transition period between CKD to end stage kidney disease (ESKD) is associated with high mortality – and those without adequate preparation have also been shown to have higher and longer hospitalization rates, more catheter hemodialysis access, lower utilization of home dialysis, lower transplantation rates and higher mortality [18, 19]. Adequate preparation and planning in the outpatient setting are crucial to avoid suboptimal initiation of kidney replacement therapy (KRT).

Here, we report the implementation and results of a quality improvement strategy to improve outpatient kidney transplant referrals in a single center academic outpatient nephrology clinic.

## Methods

### Assessment of kidney transplant referral rate

This study was performed at the Icahn School of Medicine at Mount Sinai Nephrology Fellows’ clinic, which is part of an outpatient network within the Department of Ambulatory Care of the Mount Sinai Health System. The clinic is staffed by 10 nephrology fellows (1st and 2nd training year) who are supervised by 3 attending nephrologists. Each week, at least 50 adult patients are seen.

To assess the clinic’s baseline kidney transplantation referral rate, all patients between the ages of 18 and 75 years old with eGFR ≤20 ml/min/1.73 m^2^ (2 values ≥90 days apart) seen over a three-month period (September 1, 2016 to November 30, 2016) at the clinic were identified. eGFR was calculated using the Chronic Kidney Disease Epidemiology (CKD-EPI) formula in the electronic medical record. These charts were manually reviewed to look for evidence of kidney transplant referral which included any encounter in the electronic medical record in the patient’s chart with Mount Sinai Hospital kidney transplant clinic. In addition, documentation was reviewed to look for evidence of referral to kidney transplantation in physician’s notes.

### Root cause analysis

**Fig. 1:**
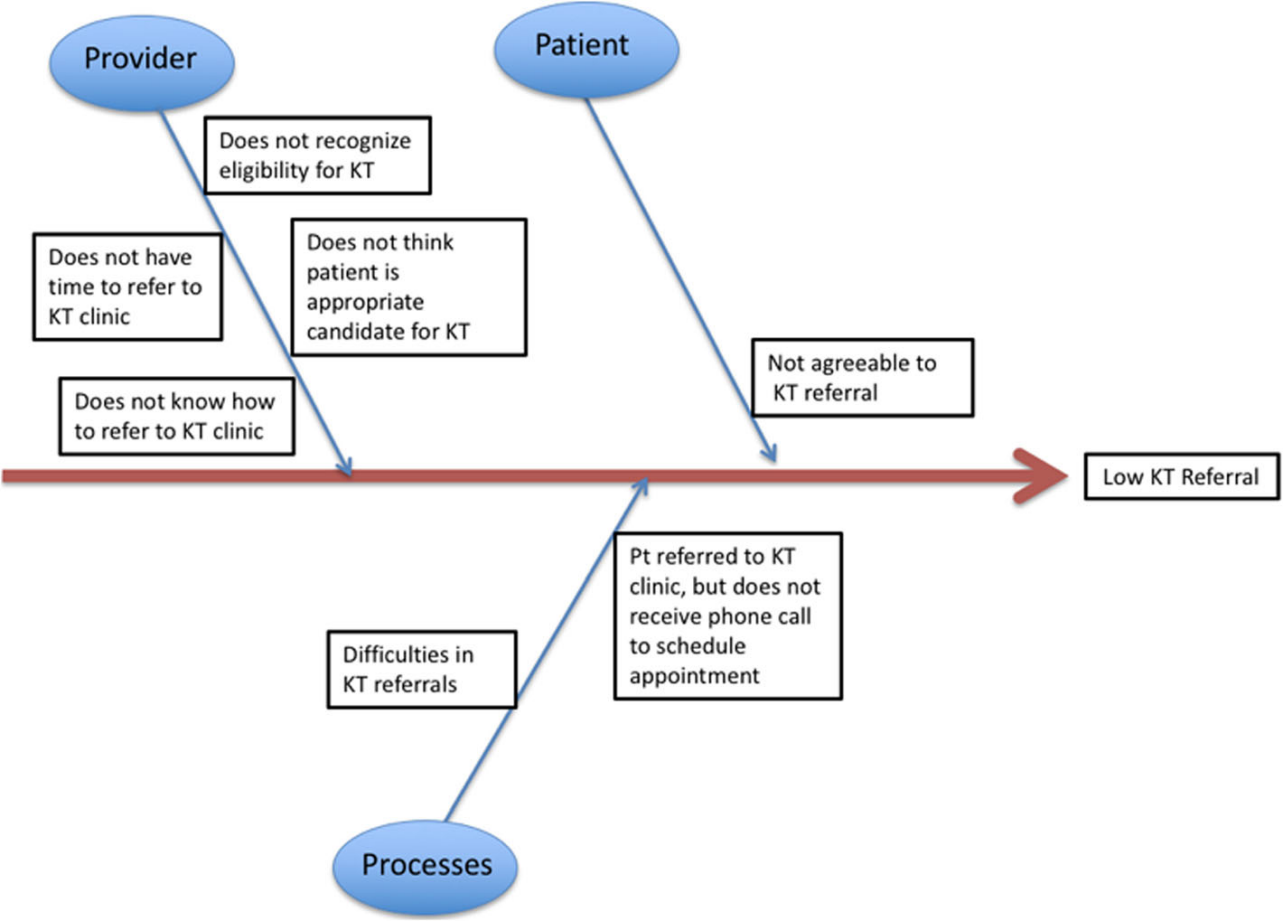
Fishbone Diagram and Root Cause Analysis of Low Kidney Transplantation Referral Rate

A cause and effect diagram, also known as a fishbone or Ishikawa diagram [20], was used to explore and display possible causes of lack of referral to kidney transplantation and ultimately identify areas for improvement (Fig. 1). Both trainees and attending nephrologists were informally surveyed and asked about perceived barriers to kidney transplantation referrals.

### Strategy to improve kidney transplant referrals

**Fig. 2:**
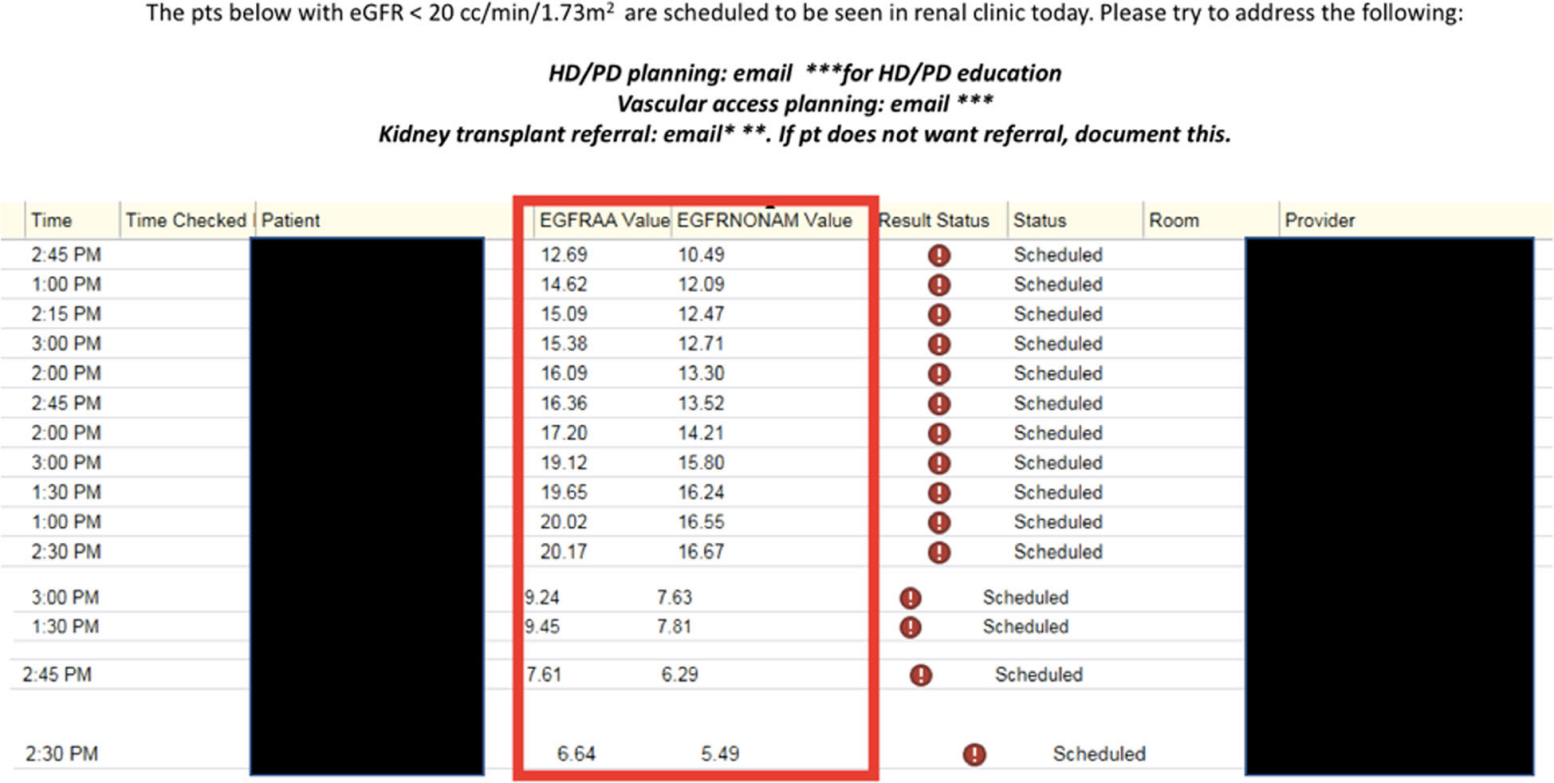
Document for Physicians’ Identifying Patients Eligible for Kidney Transplantation Referral

We hypothesized that a quality improvement intervention targeting the nephrologist’s role in the transplant referral process would lead to an improvement in rates of referral for patients who met criteria for kidney transplant evaluation. Our intervention began on July 1, 2017. First, we implemented the addition of a column in the electronic medical record which clearly displayed the patient’s last estimated glomerular filtration rate (eGFR) in the patient clinic schedule. The eGFR appeared next to the patient’s name and time of scheduled visit (Fig. 2).

In addition, each nephrology fellow was given a document prepared by a nephrology fellow “leader” listing his/her patients with an eGFR < 20 ml/min/1.73 m^2^ who were scheduled to be seen that day as a reminder to discuss kidney transplantation before the start of each clinic session. This document also listed an e-mail address for a kidney transplant clinic liaison to whom referral information could be directly sent. Finally, annual education sessions to discuss the importance of timely kidney transplant referral were held with nephrology fellows and the attendance to these sessions was greater than 80% of fellows.

A 3 month delay after the intervention began allowed time to educate fellows. The assessment of the kidney transplantation rate was repeated from 09/01/17 to 11/30/17 and compared to the pre-intervention rate. Data addressing subsequent evaluation by the kidney transplant clinic, listing on the deceased donor kidney transplant wait list, or living donor kidney transplantation were not collected.

### Statistical analysis

Normally distributed continuous variables are described using means and standard deviations; median and 25th–75th percentiles are used otherwise. Categorical variables are described as percentages. The outcomes were analyzed by Chi-square test and Student’s t-test, as appropriate; Fisher exact test was performed.

## Results

At baseline, 54 unique patients with eGFR ≤20 ml/min/1.73 m^2^ (2 values ≥90 days apart) were identified who were seen in the Clinic between July 1, 2016 and September 30, 2016. Their demographics are described in Table 1. 29.6% (16) eligible patients were referred for kidney transplantation evaluation. 69.5% (37) of these patients were not referred for kidney transplant evaluation. 46.3% (25) did not have documentation regarding kidney transplant in the nephrologist’s most recent note. For those patients whose clinical documentation mentioned kidney transplantation but referral was not performed, representative examples include “given age and stability of kidney function, will defer referral to transplant clinic at this time”, “patient states that kidney transplant has been discussed in the past and she does wish to pursue this” , “given acute medical issues and co-morbidities, will defer referral to kidney transplant clinic at this time.”

**Table 1:** Demographics of Patients, Pre-Intervention and Post-Intervention

	Pre-Intervention (*n* = 54)	Post-Intervention (*n* = 66)	*p*-value
Age (Mean, STD)	63.7 (15.1)	61.2 (16.2)	0.17
Female (%)	63.1	68.2	0.10
Race (%)			0.26
African American	39.6	41.5	
Caucasian	6.3	4.6	
Hispanic	33.3	28.8	
Unknown/Other	20.8	24.2	
DM (%)	58.2	56.1	0.14
HTN (%)	82.1	87.9	0.08
Estimated glomerular filtration rate (eGFR, Mean, STD)	11.5 (4.9)	11.7 (4.3)	0.85
Transplant Referrals (%)	29.6	60.6	< 0.001

Following the intervention, 66 unique patients (Table 1) met criteria for eligibility for kidney transplant evaluation between September 1, 2017 and November 30, 2017. Kidney transplant referrals increased to 60.6% (*p* <  0.001).

## Discussion

This pilot implementation study of a strategy to improve outpatient kidney transplant referrals showed that a free, simple, scalable intervention can significantly improve kidney transplant referrals in the outpatient setting. This intervention targeted the nephrologist’s role in the transplant referral, and facilitated the process of patient recognition and performing the referral itself without significantly interrupting the workflow. We believe that one important reason for lack of referral to kidney transplantation is the physician’s perception that the patient is not eligible for kidney transplant. Other reasons that that patient’s may not have been referred to kidney transplant include loss to follow up, the patients’ personal preference to decline kidney transplant evaluation, and inadequate time during the routine visit. Late referrals to nephrologists may also play a role in delayed kidney transplant referrals. For example, urgent preparation for dialysis make take precedence if a patient with CKD is establishing care with the nephrologist late in the course of the disease. Factors that have been attributed to late referrals include the PCP as well as patient and healthcare factors. Though timely referrals to both nephrologists and transplant nephrologists may increase the likelihood of preemptive kidney transplantation and avoidance of dialysis modalities, earlier referrals may also allow time for enhanced preparation for dialysis modalities if needed.

A similar approach to our intervention can be applied in outpatient nephrology clinics to encourage timely preparation for both dialysis and evaluation for kidney transplant. Of note, timely referral to a nephrologist is also critical to optimize outcomes for patients with CKD [21]. Data from the Australian ESKD registry revealed that patients who were referred later to a nephrologist were less likely to be put on the waiting list (odds ratio 0.49, 95% confidence interval (CI) 0.41–0.59). or given a transplant (hazard ratio 0.65, 95% CI 0.55–0.77 [18].

Strengths of the quality improvement intervention include simplicity, scalability, and generalizability for application to diverse nephrology ambulatory settings across electronic health record vendors. In addition, beyond increasing referrals to kidney transplantation, we believe that medical documentation and goals of care discussions also improve as physicians were trained to raise transplantation more frequently. The eGFR < 20 ml/min/1.73m^2^ threshold selected for the intervention is consistent with current UNOS policy that is applicable throughout the US [22]. The design could also be implemented with a different threshold, for example eGFR < 25 ml/min/1.73m^2^, since centers may encourage earlier referral. Linkage of an e-consult referral to the kidney transplantation clinic may also improve user friendliness and efficacy of our intervention.

Our intervention is a tool to address disparities in access to kidney transplantation, since preliminary success was achieved in a population enriched with African Americans and Hispanics. Existing data have shown that minoritized groups such as African American and Hispanics are referred later for transplantation in their ESKD course. Proposed contributors include patient preferences, clinician biases, poor patient education, low socioeconomic status, more frequent contraindications to transplantation, inadequate health insurance, and immunologic factors [23]. Although the intervention clinic nephrology care was primarily delivered by fellows, the intervention could be applied to attending nephrology clinics and community clinics regardless of academic affiliation. This intervention is an example of an innovation that will contribute to delivering the promise of the Advancing American Kidney Health Initiative to improve access to kidney transplantation and home dialysis [24].

Study limitations include data capture for referral exclusively within a single institution’s transplant department, making missing data for evaluation or listing at outside institutions possible. This limitation potentially only underestimates the impact of the quality improvement intervention. It is possible that the Hawthorne Effect biased the results of our study, though the details of this quality improvement study were not explicitly explained to the physicians. Future analyses will demonstrate the robustness of our intervention. Large urban academic center adult population with both a nephrology fellow’s clinic and transplant center within the same institution may limit generalizability to certain settings, particularly those in which the referring clinic and transplant center are in different locations.

## Conclusions

We describe a free, simple, scalable intervention that can significantly improve kidney transplant referrals in the outpatient setting. As kidney transplantation is the preferred treatment for ESKD, this intervention has the potential to lead to improved patient outcomes. Next steps include further investigation to study the impact of early referral to kidney transplant centers on preemptive and living donor kidney transplantation as well as successful waitlisting.

## Data Availability

The datasets generated and/or analyzed during the current study are not publicly available because the data is used internally for quality improvement processes currently, but are available from the corresponding author on reasonable request.

## Abbreviations

ESKD: End stage kidney disease
CKD: Chronic kidney disease
eGFR: estimated glomerular filtration rate
